# miR-146a attenuates apoptosis and modulates autophagy by targeting TAF9b/P53 pathway in doxorubicin-induced cardiotoxicity

**DOI:** 10.1038/s41419-019-1901-x

**Published:** 2019-09-11

**Authors:** Jian-An Pan, Yong Tang, Jian-Ying Yu, Hui Zhang, Jun-Feng Zhang, Chang-Qian Wang, Jun Gu

**Affiliations:** 1 Department of Cardiology, Shanghai Ninth People’s Hospital, Shanghai Jiaotong University School of Medicine, Shanghai, People’s Republic of China; 2 Department of Hematology, Shanghai Ninth People’s Hospital, Shanghai Jiaotong University School of Medicine, Shanghai, People’s Republic of China

**Keywords:** Apoptosis, Macroautophagy, Toxicology, Cardiomyopathies

## Abstract

Clinical therapy of doxorubicin (DOX) is limited due to its cardiotoxicity. miR-146a was proved as a protective factor in many cardiovascular diseases, but its role in chronic DOX-induced cardiotoxicity is unclear. The objective of this study was to demonstrate the role of miR-146a in low-dose long-term DOX-induced cardiotoxicity. Experiments have shown that DOX intervention caused a dose-dependent and time-dependent cardiotoxicity involving the increased of apoptosis and dysregulation of autophagy. The cardiotoxicity was inhibited by overexpressed miR-146a and was more severe when miR-146a was downgraded. Further research proved that miR-146a targeted TATA-binding protein (TBP) associated factor 9b (TAF9b), a coactivator and stabilizer of P53, indirectly destroyed the stability of P53, thereby inhibiting apoptosis and improving autophagy in cardiomyocytes. Besides, miR-146a knockout mice were used for in vivo validation. In the DOX-induced model, miR-146a deficiency made it worse whether in cardiac function, cardiomyocyte apoptosis or basal level of autophagy, than wild-type. In conclusion, miR-146a partially reversed the DOX-induced cardiotoxicity by targeting TAF9b/P53 pathway to attenuate apoptosis and adjust autophagy levels.

## Introduction

Doxorubicin (DOX), an anthracycline, was discovered in the 1960s as a breakthrough in the field of cancer chemotherapy and still be the cornerstone of today’s various chemotherapy regimens. However, cardiovascular complications are the main problems that restrict its widespread application which show dose-dependent myocardial toxicity, eventually leading to irreversible congestive heart failure^1–3^. The mechanisms of DOX-induced myocardial injury include cardiomyocyte apoptosis^4,5^, atrophy^6^, autophagy disorder^4,7^, oxidative stress^8,9^, etc. But the imbalance between apoptosis and autophagy still plays an important role in it. Apoptosis is accomplished by programmed death when cells are subjected to external stress, and autophagy maintains cell homeostasis by isolating damaged organelles and delivering them to lysosomal for degradation. Although many studies have shown that the use of some interventions after DOX chemotherapy can partially reverse myocardial injury^4,10^, how to detect cardiotoxicity and improve the myocardial condition in time remains to be explored.

MicroRNAs (miRNAs) are a class of endogenous, 22-nucleotide noncoding RNAs that regulate post-transcriptional regulation of target gene expression by binding to the 3′ untranslated region (3′-UTR)^11^. It was originally reported that miR-146a targets IRAK1 and TRAF6 as negative regulators of innate immune responses^12^. In the subsequent studies, its role in cardiovascular disease is slowly emerging, such as atherosclerosis^13–15^, myocardial infarction^16^, ischemia-reperfusion injury^17^, and diabetic cardiomyopathy^18^. The mechanism of myocardial protection involves many aspects including anti-apoptosis^16,19^, inhibition of inflammation^13,14,17^, and improvement of fibrosis to delay myocardial remodeling^18^. Even hope to become a biomarker for various cardiovascular diseases^20,21^, although more clinical trials need to be implemented to prove it. The role of miR-146a in DOX-induced cardiomyopathy, although mentioned, only discussed its effect on acute myocardial injury^22^. Clinically, most patients experience myocardial damage after long-term chemotherapy of DOX, therefore, the role of miR-146a in small doses of chronic DOX myocardial toxicity still needs further discussion.

Tumor suppressor protein (P53), also known as the cell gatekeeper, is activated in response to various stresses in a variety of heart diseases. Once activated, P53 produces a complex network of signals to induce apoptosis and regulate autophagy^23^. TAF9b, one of the TATA-binding protein (TBP) associated factors (TAFs), acts as the coactivator of P53^24^. Ability to bind to P53 protein and maintain its stability, TAF9b has been involved in the cellular process by regulating P53^25,26^.

Because miR-146a is abundantly expressed in the myocardial tissue and TAF9b has potential miR-146a-binding sites in the 3′UTR. Herein, we demonstrated that miR-146a affects the stability of P53 by targeting TAF9b, thereby inhibiting apoptosis and improving autophagy in the process of chronic DOX-induced myocardial toxicity.

## Materials and methods

### Animal experiments

The study was conducted in strict accordance with the recommendations of the “Guidelines for the Care and Use of Laboratory Animals”. All experiments were approved by the Animal Experiment Ethics Committee of Shanghai ninth people’s hospital. Male miR-146a deficient (miR-146a^−/−^) obtained from the Jackson Laboratory (https://www.jax.org/strain/016239) and wild-type (WT) genetic background control C57BL/6 mice obtained from Shanghai Model Organisms Center (Shanghai, China) were kept in sterilized filter top cages with controlled humidity and a 12-h day/night cycle at 22 °C. Standard mouse chow and tap water were provided ad libitum. Experimental manipulation was initiated with 8-week-old mice weighing 18–22 g. First, WT mice were treated with DOX (i.p. 5 mg/kg; MedChem Express, USA) once a week for 4 weeks and kept for another 2 weeks to make sure that the DOX was fully absorbed and worked. Their blood and hearts were collected in different time points (from day 0 to day 42) to determine the change of miR-146a under DOX stimulation (Fig. 5a). Then, mice were assigned to one of four groups randomly: saline-treated WT group, DOX-treated WT group, saline-treated miR146a^−/−^ group and DOX-treated miR146a^−/−^ group. Mice were given DOX (i.p. 5 mg/kg) or an equivalent volume of saline for once a week for 4 weeks. All mice were kept for another 2 weeks (Fig. 5d). All animals were weighed during the treatment. At day 28 and day 42, echocardiography was implemented and ventricular sample tissues were obtained from mice euthanized using deep isoflurane (5%) anesthesia, rinsed in ice-cold phosphate buffer saline and snap-frozen in liquid nitrogen.

### Echocardiography

Transthoracic echocardiography was performed using the UBM system (Vevo 770, Visualsonics, Canada) equipped with a 40 MHz mechanical transducer. Mice were anesthetized and maintained under 1–3% isoflurane during the procedure. Echocardiographic measurements were performed by a blinded investigator and were conducted at the mid-papillary muscle level, as guided by two-dimensional short-axis images. Fractional shortening and EF were measured and calculated with Vevo Analysis software (version3.0.0).

### Cell lines and culture conditions

The human AC16 cell line, even without the formation of fully differentiated cardiac muscle cells, develops many biochemical and morphological features characteristic of cardiomyocytes^27^, purchased from the American Tissue Culture Centre (ATCC, USA). Cells were maintained in medium composed of Dulbecco’s modified Eagle’s medium (DMEM) (Hyclone, USA) supplemented with 10% fetal bovine serum (Sigma, USA) and 1% penicillin–streptomycin solution (Hyclone, USA) at 37 °C in a humidified incubator containing 95% air and 5% CO2. The media was refreshed every 3 days and replaced with serum-free DMEM for 12 h when cells cultured at about 80% confluence. DOX treatment was used at different times and concentrations subsequently.

### Cell transfection

For in vitro overexpression and downregulation of miR-146a, miR-146a mimics and inhibitors (Ribobio, China) were used. For knockdown of TAF9b, a small interfering RNA (siRNA) molecule (siTAF9b) (GenePharma, China) were synthesized. The target sequences were listed in Tables S1 and S2. A random sequence molecule was synthesized as negative control respectively. AC16s were seeded at a density of 1 × 10^5^ cells/ml in a well plate containing growth medium without antibiotics and incubated overnight. Lipofectamin^TM^ 3000 (Invitrogen, USA) was used according to the manufacturer’s instructions for 48 h to transfect in OPTI-MEM reduced serum medium (Gibco, USA). The effects of these interventions were evaluated by FAM fluorescence and real-time PCR. The following experimental treatments were performed after transfection.

### Western Immunoblot

To obtain total protein extracts, cultured AC16s or frozen cardiac tissues were lysed at 4 °C in radioimmunoprecipitation (RIPA) buffer with cocktail protease inhibitor (Beyotime Biotechnology, China). Tissue homogenates or cell lysates were clarified by centrifugation at 12,000 rpm for 15 min at 4 °C and the supernatant protein concentration was determined using the bicinchoninic acid method (Beyotime Biotechnology, China). Proteins (20 μg) were size-fractionated by sodium dodecyl sulphate polyacrylamide gel electrophoresis and transferred onto Immobilon polyvinylidene difluoride membranes. The membranes were blocked for 2 h with 5% Difco^TM^ Skim Milk (BD Biosciences, USA) and then probed overnight at 4 °C with primary antibodies: GAPDH mouse polyclonal antibody (1:5000, 60004–1-Ig, Proteintech), P53 rabbit polyclonal antibody (1:5000, 10442–1-AP, Proteintech), TAF9b rabbit monoclonal antibody (1:1000, 45238, Signalway Antibody), Bax rabbit monoclonal antibody (1:1000, 14796, Cell Signaling Technology), Caspase-3 rabbit monoclonal antibody (1:1000, 9665, Cell Signaling Technology), Beclin-1 rabbit monoclonal antibody (1:1000, 3738, Cell Signaling Technology), LC3B rabbit monoclonal antibody (1:1000, 3868, Cell Signaling Technology) and Bcl-2 rabbit monoclonal antibody (1:1000, ab59348, Abcam), followed by secondary antibody for a hour with a 1:30,000 dilution of DylightTM800 4XPEG-conjugated goat anti-rabbit IgG (H + L) or goat anti-mouse IgG (H + L), (Cell Signaling Technology, USA). The results were visualized by Odyssey Infrared Imaging System.

### RNA preparation and analysis

Total RNA was extracted from cells, cardiac tissues and serum using the TRIzol reagent (Invitrogen, USA), an equal amount of the external reference gene cel-miR-39 (Ribobio, China) was added when it came to serum, and reverse-transcribed into cDNA using PrimeScript™ RT reagent Kit (Takara, Japan). Next, the cDNA was quantitatively amplified using TB Green Premix Ex Taq II (Takara, Japan). Real‐time PCR was conducted in triplicate using an Applied Biosystems 6Flex. The sequences of the forward and reverse primers used for amplification are shown in supplementary material Table S3. The results for the expression of TAF9b were presented relative to the expression of the GAPDH gene and relative miR-146a expression was normalized to the expression of U6 small nuclear RNA (snRNA) in cells and tissues or cel-miR-39 in serum by the Delta–Delta Ct method.

### Dual-luciferase reporter assay

PmirGLO-TAF9b plasmids were constructed containing either wide type or mutant 3′ untranslated region (UTR) sequence of TAF9b mRNA (BioLink, China). The plasmids were transfected into HEK293T cells with pre-miR-146a plasmids or corresponding control pcDNA6.2-GW using Lipofectamine^TM^ 3000 reagents in triplicate. Cells were harvested at 48 h after transfection. The activities of firefly and Renilla luciferases were measured sequentially by a Dual-Glo Luciferase Assay System (Promega, USA). Relative luciferase activities were quantified by normalizing Renilla luciferase values to firefly values.

### CCK-8 cell viability assay

Cell Counting Kit-8 (CCK-8) was obtained from Dojindo (Dojindo, Japan). AC16 cells, pretreated in different ways, were plated (5000 cells/well) into 96-well plates and cultured in the growth medium. At the indicated time points, the viability of cells in triplicate wells was measured using the absorbance at 450 nm.

### TUNEL-staining analysis

TUNEL apoptosis detection kit (Alexa fluor 488) (Yeasen, China) was used according to the manufacturer’s protocol to detected nuclear fragmentation. Cardiac tissue sections were deparaffinized in xylene and rehydrated in aqueous solutions with decreasing alcohol content, followed by a wash in PBST (1 × PBS with 0.5% Tween 20, pH 7.4). For the TUNEL staining of AC16s, cells were rinsed with PBS, fixed with 4% paraformaldehyde. Both tissue sections and cells were incubated by proteinase K for 5 min then were labeled using the TdT reaction and incubated for 1 h at 37 °C. After that, Hoechst 33342 (Beyotime Biotechnology, China) was used for nuclear staining. The apoptotic cells were then visualized under a fluorescence microscopy (Nikon, Japan) and counted from five randomly selected fields by image J.

### Determination of autophagy flux

Autophagy was determined by the detection of the fluorescence microscopic detection of the formation of the autophagosomes in cells transfected with pSELECT-LC3-GFP (Sigma-Aldrich, USA). AC16 cells were cultured and a plasmid expressing GFP-LC3 was used for transduction at 37 °C for 48 h. Next, the transfected cells were treated with DOX and other interventions. Cells were fixed with 4% paraformaldehyde and Hoechst 33342 was used for nuclear staining. Finally, the autophagy flux was analyzed using a fluorescence microscopy (Nikon, Japan) and fluorescence intensity is calculated to represent the strength of the autophagic flow by image J.

### Co-immunoprecipitation (Co-IP)

Co-IP experiments were performed to examine the interaction between P53 and TAF9b. The cells were lysed with Co-IP Lysis Buffer (20 mM Tris [pH = 8.0], 150 mM NaCl, 0.5% NP-40, 10% Glycerol, 2 mM EDTA) with Cocktail protease inhibitor at 4 °C for 30 min, and centrifuged at 12,000 rpm for 10 min. The supernatant (300 μg of total proteins) were pre-incubated with 2 μg IgG of the same isotype as the primary antibody to block nonspecific combination, followed by incubation with 5 μg primary rabbit anti‐P53 antibody or anti-TAF9b antibody overnight at 4 °C. The normal IgG was used for comparison. After that, Protein G-agarose beads (Roche, Germany) were then added and slowly oscillated at 4 °C for 4–6 h to precipitate the antibody complexes and the precipitates were then subjected to regular Western blot analysis as described above.

### Flow cytometry

FITC Annexin V Apoptosis Detection Kit (BD Biosciences, USA) was used to determine the rate of apoptosis among cells. Treated cells were harvested, washed with precooled PBS for twice, resuspended in Annexin V binding buffer and stained with FITC Annexin V and propidium iodide (PI). Samples were analyzed on the CytoFLEX S (Beckman, USA). CytExpert was used to calculate the percentage of cells positive for Annexin V and PI.

### Hematoxylin-eosin (HE) staining

The myocardial tissue was fixed in 4% paraformaldehyde, embedded in paraffin, sectioned (5 μm), de-waxed and hydrated, and stained with HE. The form, size, and arrangement of cardiomyocytes were observed by optical microscope (Nikon, Japan).

### Transmission electron microscopy (TEM)

TEM was used to observe mitochondrial state and autophagic vacuoles of tissues. Freshly excised myocardial tissues were quickly cut into 1 mm cubes fixed overnight in 2.5% glutaraldehyde and then post-fixed with 1% osmium tetroxide, dehydrated through a graded ethanol series and embedded in epoxy resin. Ultrathin sections (70 nm) were collected and double stained with uranyl acetate and lead citrate. Finally, the sections were observed by electron microscopy.

### Statistical analysis

Statistical analysis was performed by using SPSS 22.0 software. All data were presented as mean ± SD at least three independent experiments. The differences between all measured values were assessed by Student’s *t* test for two groups, one-way ANOVA followed by Bonferroni post hoc test for multiple groups, and a parametric generalized linear model with the relationship between circulating miR-146a levels and BNP. *P* values <0.05 were considered significant.

## Results

### DOX amplified apoptosis and disturbed autophagy in AC16s

**Fig. 1:**
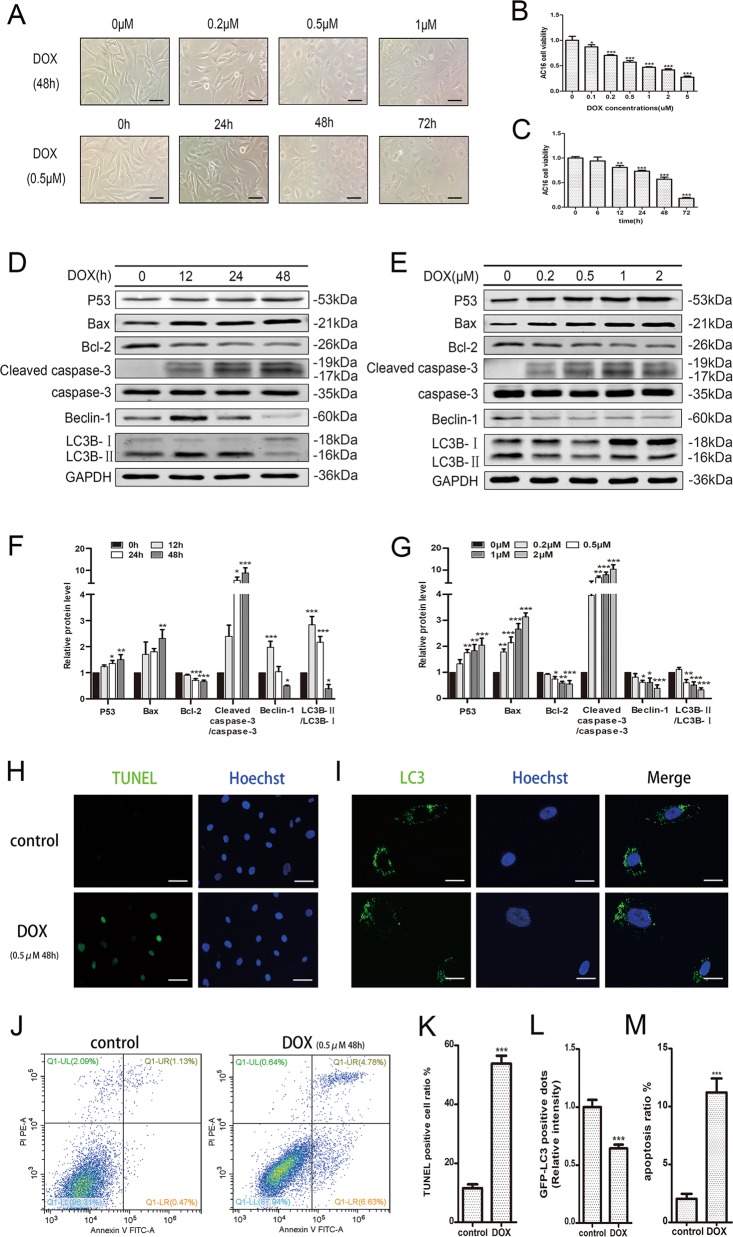
DOX amplified apoptosis and disturbed autophagy in AC16s. AC16 cells were treated with different concentrations of DOX for 48 h or different times for 0.5 μM. **a** The morphological changes of cells after DOX intervention were observed under a microscope. Scale bar indicated 50 μm. **b**, **c** Cell viability after doxorubicin treatment was detected by CCK-8 cell viability assay and normalized to control. **d**, **e** Apoptosis and autophagy-related proteins of AC16s were detected after DOX treatment by western blot. **f**, **g** The relative protein expression levels were determined by densitometric analysis. GAPDH was included in the analysis as a control. **h** TUNEL staining analysis was used to detected nuclear fragmentation after DOX intervention at 0.5 μM for 48 h. Scale bar indicated 50 μm. **i** Representative fluorescence microscopy images of GFP-LC3 transfected cells treated as indicated. Scale bar indicated 20 μm. **j** Apoptosis was then analyzed by staining with propidium iodide (PI, *y*-axis) and annexin V-FITC (*x*-axis). **k** The percentage of TUNEL positive cells in each group according to Hoechst nuclear staining was indicated. **l** The relative GFP-LC3 positive dots were calculated according to the fluorescence intensity. **m** The percentage of PI-positive cells in each quadrant were indicated to represent the apoptotic rate of cells. Mean ± SD of three independent experiments. *P < 0.05, **P < 0.01, ***P < 0.001

To explore the effects of DOX on cardiomyocytes, we used a series of concentration gradients for 48 h and time gradients for 0.5 μM of DOX to intervene in AC16s. Cells were treated after a 12-h serum deprivation cultivate. Obviously, as the concentration or treated time of DOX increased, the morphology of the cells slowly shrank from the intact fusiform to round, the cytoplasm became dense, and finally ruptured (Fig. 1a). The cell viability significantly declined detected by CCK-8 cell viability assay and had dropped by about 50% with DOX at 0.5 μM for 48 h (Fig. 1b, c). Western blot analysis of the expression levels of apoptosis and autophagy-related proteins showed that DOX increased the expression of pro-apoptotic factors P53, Bax and cleaved caspase-3, whereas Bcl-2, an anti-apoptotic indicator, decreased after DOX treatment (Fig. 1d–g). Interestingly, autophagy-related proteins such as Beclin-1 and LC3B-II/LC3B-I had a transient increase in the early stage of DOX intervention, decreased at 48 h, and became more pronounced as the concentration increased (Fig. 1d–g).

For further proof, we took the DOX treatment at 0.5 μM for 48 h. TUNEL-staining detection of DNA fragmentation revealed that DOX significantly increased the rate of apoptosis in AC16 cardiomyocytes (Fig. 1h, k), consistent with the results obtained by Annexin V/PI stained flow cytometry (Fig. 1j, m). As for autophagy, GFP-LC3, which bound to autophagic vesicles, suggesting that autophagy flux was inhibited by DOX so that damaged contents cannot be cleaned up in time (Fig. 1i, l). In summary, DOX-mediated myocardial toxicity was dose and time-dependent, and its mechanism was related to increasing apoptosis and disturbing normal autophagy.

### MiR-146a was increased by DOX stimulated and attenuated DOX-induced cardiotoxicity in AC16s

**Fig. 2:**
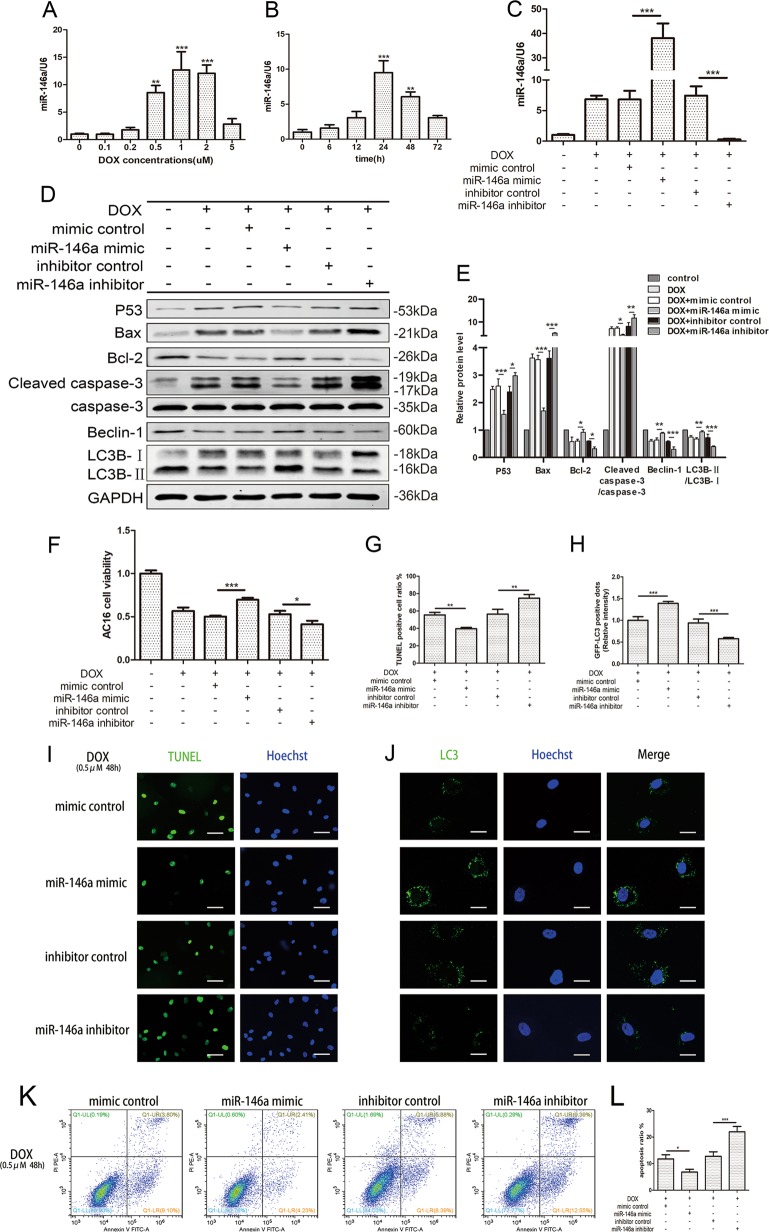
MiR-146a was increased by DOX stimulated and attenuated DOX-induced cardiotoxicity in AC16s. **a**, **b** The expression of miR-146a in AC16s were detected after DOX incubated with different concentrations or different times by qRT-PCR normalized to U6. Hereafter, cells were transfected with miR-146a mimics (50 nM) and inhibitors (100 nM) to overexpress or inhibit the expression of miR-146a, subsequently treated with DOX at 0.5 μM for 48 h. **c** qRT-PCR was used to verify transfection efficiency. **d**, **e** Apoptosis and autophagy-related proteins were detected by western blot after transfection and the relative protein expressive were determined normalized to GAPDH. **f** Cell viability after transfection was detected by CCK-8 cell viability assay and normalized to control. **g**, **i** TUNEL staining analysis was used to detecting nuclear fragmentation after transfection. Scale bar indicated 50 μm. The percentage of TUNEL-positive cells in each group according to Hoechst nuclear staining was indicated. **h**, **j** Representative fluorescence microscopy images of GFP-LC3 transfected cells treated as indicated. Scale bar indicated 20μm. The relative GFP-LC3 positive dots were calculated according to the fluorescence intensity. **k**, **l** Apoptosis was analyzed by staining with propidium iodide (PI, *y*-axis) and annexin V-FITC (*x*-axis). The percentage of PI-positive cells in each quadrant were indicated to represent the apoptotic rate of cells. Mean ± SD of three independent experiments. *P < 0.05, **P < 0.01, ***P < 0.001

Since studies have shown that miR-146a was abundantly expressed in cardiomyocytes and played an important role in a great number of heart diseases^15,18^. We examined the expression of miR-146a in AC16 cardiomyocytes after DOX incubated and found that the expression of miR-146a showed a concentration-dependent and time-dependent increase, and decreased to a certain extent (Fig. 2a, b).

In order to explore whether miR-146a had a protective effect on myocardial toxicity caused by DOX, we transfected AC16s with miR-146a mimics and inhibitors before the cells were stimulated with DOX. Scrambled mimics and inhibitors served as control respectively. Detection of the expression of miR-146a after treatment with DOX at 0.5 μM for 24 h (Fig. 2c) and apoptosis and autophagy-related proteins for 48 h (Fig. 2d, e) suggested that overexpressed miR-146a inhibited apoptosis and restored autophagy. When miR-146a was inhibited, the apoptosis became more serious but the autophagy was extremely suppressed. Cell viability detected by CCK-8 was partially improved when miR-146a was pre-overexpression as well as exacerbated when was silenced (Fig. 2f). TUNEL staining (Fig. 2g, i) and flow cytometry (Fig. 2k, l) could more intuitively reflect changes in apoptosis and GFP-LC3 suggested the autophagy flux was ameliorated by elevated miR-146a (Fig. 2h, j). Taken together, these findings suggested that miR-146a had a protective effect in low-dose chronic DOX toxicity in cardiomyocytes, which was produced by reducing apoptosis and increasing autophagy flux.

### MiR-146a in AC16s inhibited DOX-induced cardiotoxicity by suppressing TAF9b/P53 pathway

**Fig. 3:**
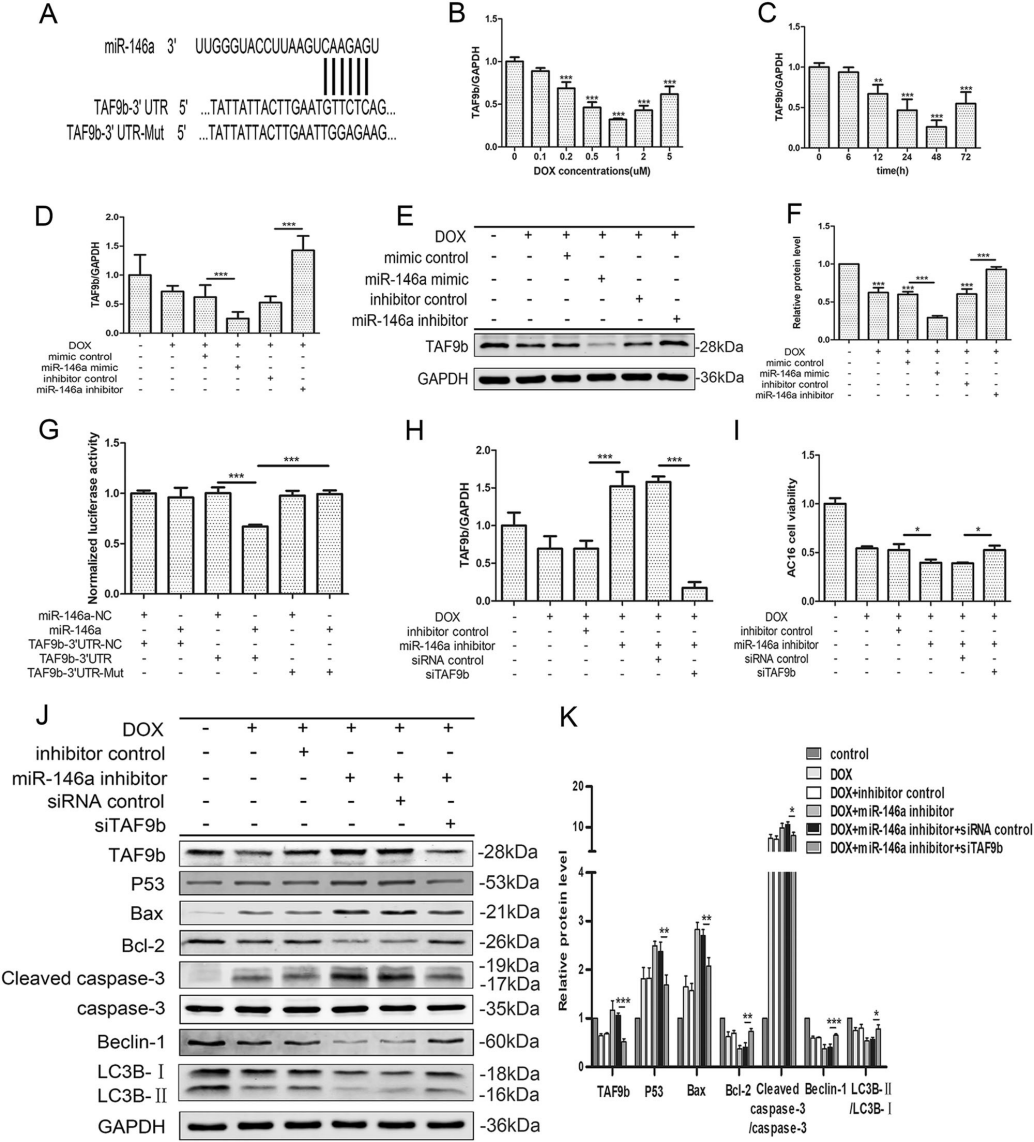
MiR‐146a in AC16s inhibited DOX‐induced cardiotoxicity by suppressing TAF9b/P53 pathway. **a** Bioinformatics analysis revealed a conserved binding site between miR-146a and TAF9b 3′‐UTR and a mutated version of the TAF9b 3′‐UTR was constructed. **b**, **c** The expression of TAF9b in AC16s were detected after DOX incubated with different concentrations or different times by qRT-PCR normalized to GAPDH. **d**–**f** The expression of TAF9b was detected in RNA level by qRT-PCR and protein level by western blot after the transfection of miR-146a mimics or inhibitors. **g** Dual-luciferase reporter assay was used to verify the specific binding between miR-146a and TAF9b in HEK293T cells. The normalized luciferase activity was significantly lower when the wild‐type TAF9b 3′‐UTR was co‐transfected with pre-miR‐146a compared with other groups. Hereafter, cells were transfected with siTAF9b (50 nM) on the basis of transfection of miR-146a inhibitors, subsequently treated with DOX at 0.5 μM for 48 h. **h** RT-PCR was used to verify transfection efficiency. **i** Cell viability after transfection was detected by CCK-8 cell viability assay and normalized to control. **j**, **k** Related proteins were detected by western blot after transfection and the relative protein expressive were determined normalized to GAPDH. Mean ± SD of three independent experiments. *P < 0.05, **P < 0.01, ***P < 0.001

To investigate the mechanism of miR-146a improving DOX-induced cardiotoxicity, bioinformatics analysis (http://www.targetscan.org/) was used to predict its possible binding genes. Overlap analyses showed that miR‐146a had a broadly conserved binding site with TAF9b (Fig. 3a). After the DOX intervention at different concentrations and different times, the RNA level of TAF9b is opposite to that of miR-146a (Fig. 2a, b, Fig. 3b, c). Then, the expression of TAF9b decreased after overexpression miR-146a and increased when miR-146a was inhibited, whether at the RNA level (Fig. 3d) or the protein level (Fig. 3e, f). For further verification, a mutated version of the TAF9b 3′-UTR was constructed and fused to the luciferase coding region (pmirGLO-h_TAF9b 3′-UTR) (Fig. 3a), Subsequently co-transfected into HEK293T cells along with pre-miR-146a or control. The normalized luciferase activity was significantly lower when the wild‐type TAF9b 3′-UTR was co-transfected with pre-miR-146a compared with other groups, confirming that miR-146a specifically targeted and suppressed TAF9b (Fig. 3g).

**Fig. 4:**
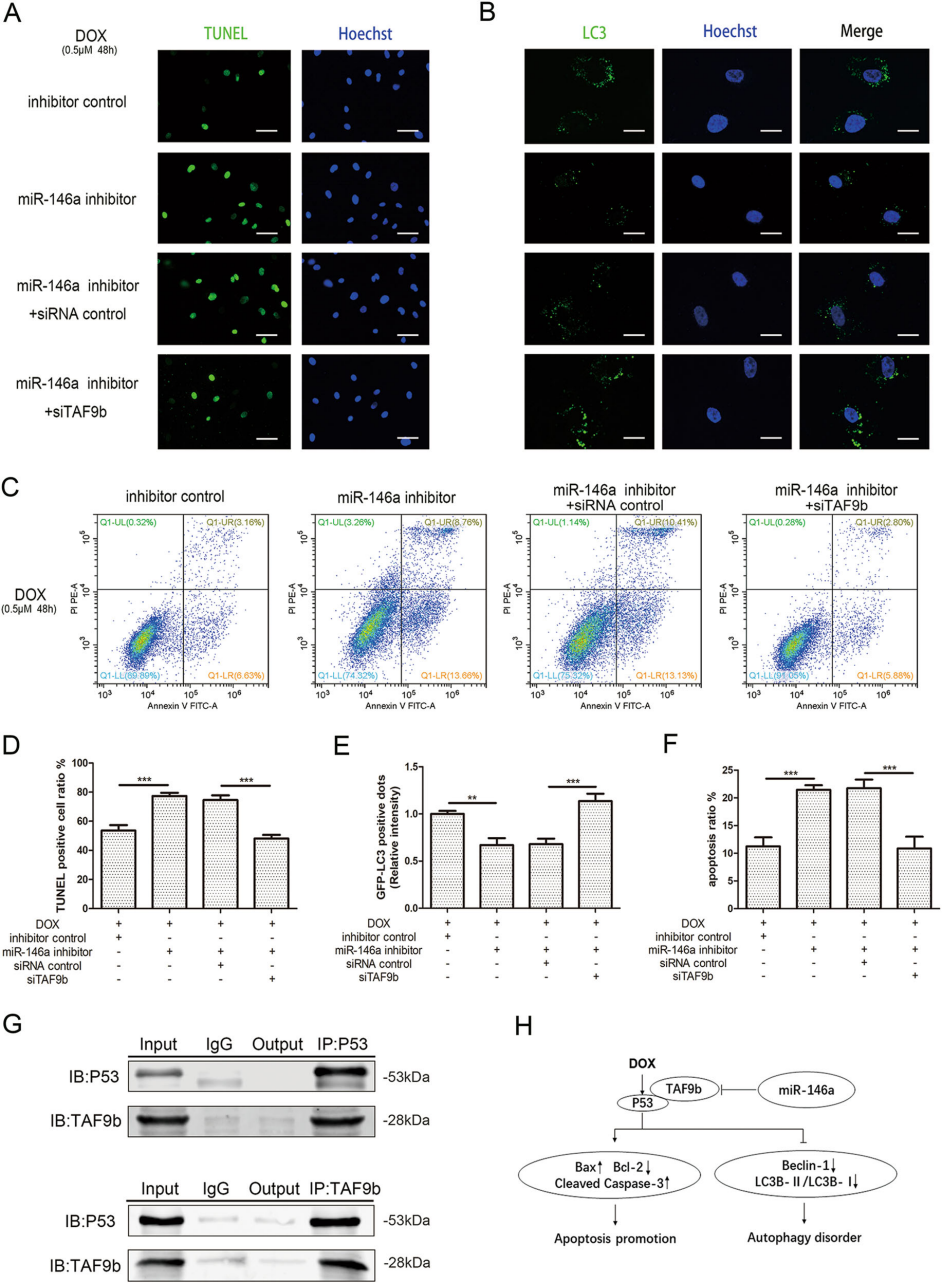
MiR‐146a in AC16s inhibited DOX‐induced cardiotoxicity by suppressing TAF9b/P53 pathway. **a**, **d** TUNEL-staining analysis was used to detected nuclear fragmentation after transfection. Scale bar indicated 50 μm. The percentage of TUNEL-positive cells in each group according to Hoechst nuclear staining was indicated. **b**, **e** Representative fluorescence microscopy images of GFP-LC3-transfected cells treated as indicated. Scale bar indicated 20 μm. The relative GFP-LC3-positive dots were calculated according to the fluorescence intensity. **c**, **f** Apoptosis was then analyzed by staining with propidium iodide (PI, *y*-axis) and annexin V-FITC (*x*-axis). The percentage of PI-positive cells in each quadrant were indicated to represent the apoptotic rate of cells. **g** Co‐IP experiments were performed to examine the interaction between P53 and TAF9b. Input represents whole cell lysate. IgG stands for isotype control. Output represents the supernatant after Co-IP. IP stands for immunoprecipitation while IB, immunoblotting. **h** Schematic illustration of the pathway of autophagy and apoptosis regulated by miR-146a in DOX-induced cardiotoxicity. Mean ± SD of three independent experiments. *P < 0.05, **P < 0.01, ***P < 0.001

Based on this, a siRNA for TAF9b was designed and co-transfected with miR-146a inhibitors to attenuate the upregulated TAF9b both in RNA level (Fig. 3h) and protein level (Fig. 3j, k). Cell viability was partially reinstated after co-transfection (Fig. 3i). Western blot suggested that the pro-apoptotic proteins such as Bax and cleaved caspase-3 were decreased while anti-apoptotic factor Bcl-2 was rise (Fig. 3j, k). TUNEL staining (Fig. 4a, d) and flow cytometry (Fig. 4c, f) demonstrated the function of siTAF9b to rescue the apoptosis of AC16s. For autophagy, Beclin-1 and LC3B-II/LC3B-I had partially restored (Fig. 3j, k) and GFP-LC3 spots increased compared to miR-146a inhibitors transfection only (Fig. 4b, e).

Studies have shown that TAF9b act as a P53 co-activator by stabilizing it and maintained P53 activity at the protein level^24^. To further verified the role of TAF9b in this progress, we certified the combination of TAF9b and P53 through Co-IP by two sides (Fig. 4g). and the expression of P53 was decreased after the transfection of siTAF9b (Fig. 3j, k). In other words, miR-146a played a corresponding protective role by targeting TAF9b, thereby inhibiting the stability and activity of P53.

### Knockout of miR-146a aggravated DOX-induced myocardial injury in vivo

**Fig. 5:**
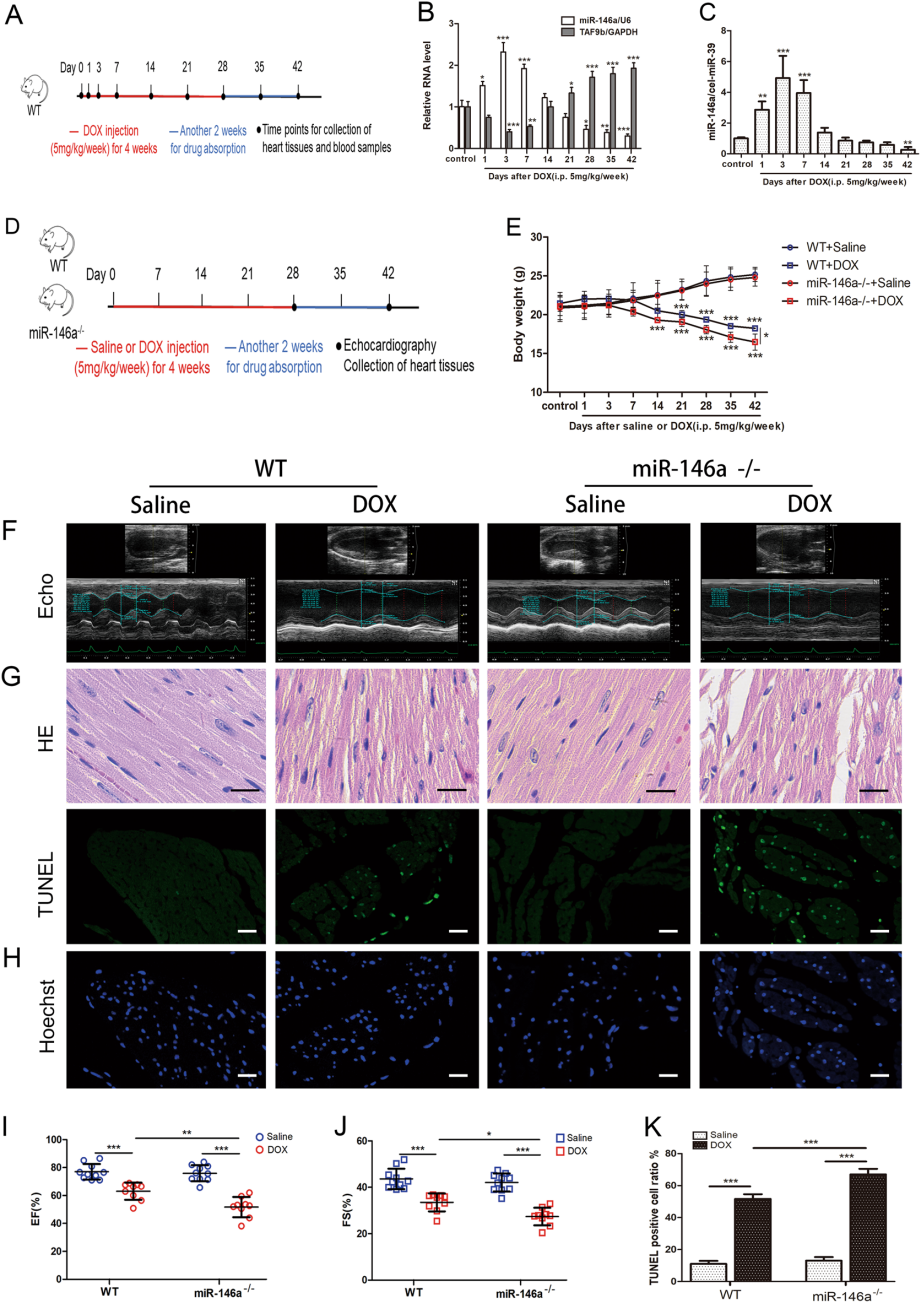
Knockout of miR-146a aggravated DOX-induced myocardial injury in vivo. **a** Male wild-type (WT) C57BL/6 mice were used and DOX (i.p. 5 mg/kg) were given for once a week for 4 weeks and kept for another 2 weeks for drugs absorption. The blood and myocardial tissues were collected in different time points. **b** The expression of miR-146a and TAF9b in myocardial tissue were detected in a series of times after DOX injected by qRT-PCR (*n* = 6). **c** The expression of miR-146a in serum was detected in a series of times after DOX injected (*n* = 6). **d** Male miR-146a deficient (miR-146a^−/−^) and WT genetic background control C57BL/6 mice were used and DOX (i.p. 5 mg/kg) or an equivalent volume of saline were given for once a week for 4 weeks, mice were kept for another 2 weeks for drugs absorption. **e** Body weight changes during administration were measured (*n* = 5). **f** Typical echocardiography at day 42 showed that DOX significantly reduced cardiac function which was more severe after miR-146a knockout. Ejection fraction (EF) and fraction shortening (FS) were shown in (**i**) and (**j**) (*n* = 10). **g** Hematoxylin-eosin (HE) staining was used to assess myocardial damage at day 42. Scale bar indicated 20 μm. **h** TUNEL staining analysis was used to detected nuclear fragmentation at day 42. Scale bar indicated 50 μm. And the percentage of TUNEL-positive cells in each group according to Hoechst nuclear staining was indicated in (**k**) (*n* = 5). *P < 0.05, **P < 0.01, ***P < 0.001

**Fig. 6:**
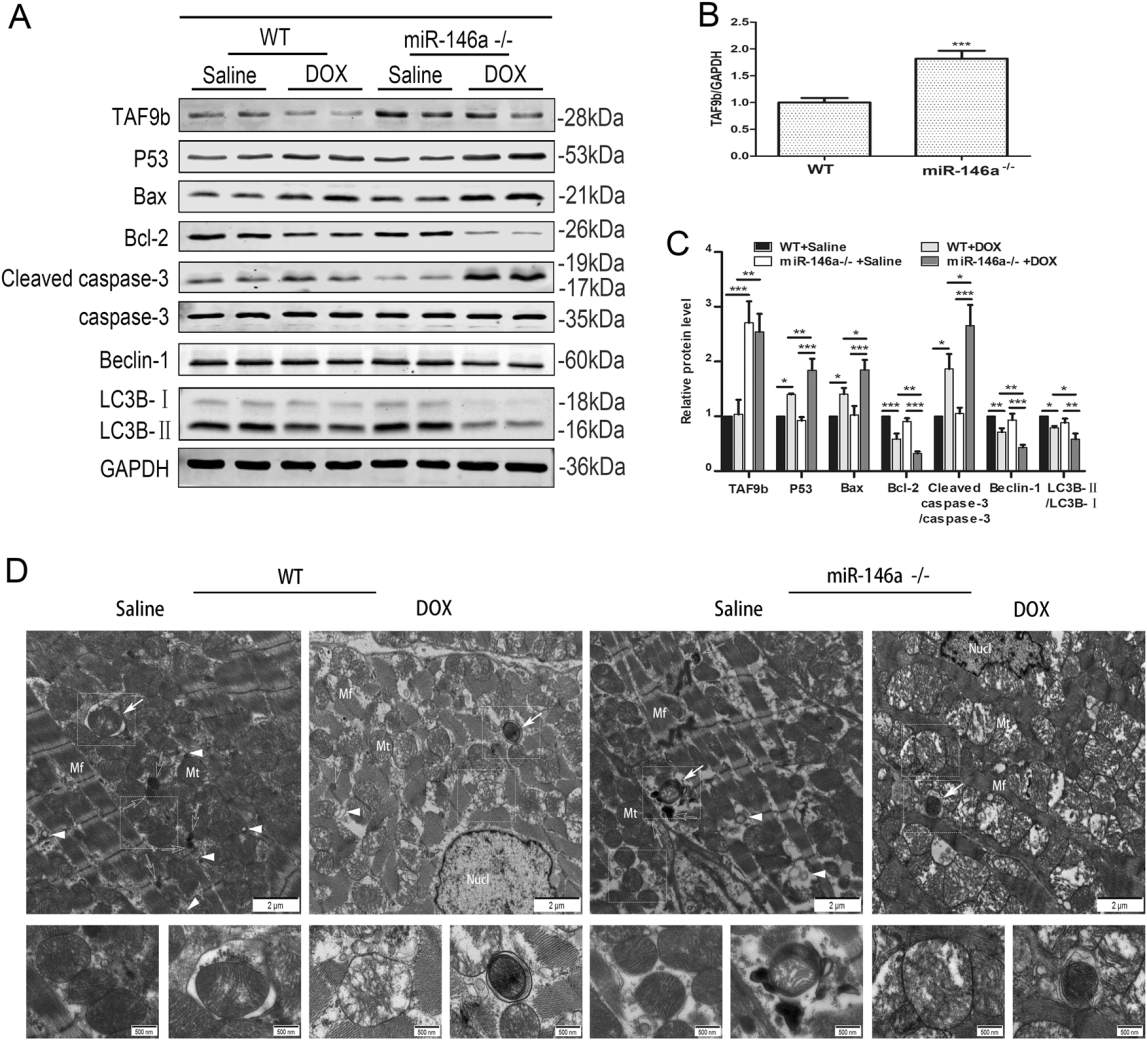
Knockout of miR-146a aggravated DOX-induced myocardial injury in vivo. **a** Related proteins of myocardial tissue were detected by western blot after DOX intervention at day 42 and the relative protein expressive were determined normalized to GAPDH in (**c**) (*n* = 5). **b** qRT-PCR was used to detected the expression of TAF9b after knockout of miR-146a. **d** Electron micrographs of autophagic vacuoles in cardiomyocytes. Hollow arrows indicated lysosomes (electron-dense spherical bodies). Solid arrowheads were instead for autophagic vacuoles. Solid arrows represented mitochondrial autophagy. Mt, mitochondria; Mf, myofibril; Nucl nucleus. *P < 0.05, **P < 0.01, ***P < 0.001

To further verify the role of miR-146a in counteracting DOX’s cardiotoxicity in vivo, male WT mice were used and DOX (i.p. 5 mg/kg/week) were treated for 4 weeks and kept for another 2 weeks to make DOX worked completely. and the myocardial tissues were collected in different time points (Fig. 5a). After the injection of DOX, the expression of miR-146a in myocardial tissue increased, peaked in day 3, lasted for a week and the compensatory increased miR-146a was slowly consumed, which was lower than normal at Day 35 and Day 42. While the mRNA level of TAF9b was the opposite (Fig. 5b). Then, the miR-146a^−/−^ mice were used. The expression of TAF9b was increased after knockout (Fig. 6a–c). Mice were given DOX or an equivalent volume of saline for once a week for 4 weeks and kept for another 2 weeks to make DOX worked completely (Fig. 5d). At the end of day 28 (Fig. S1A, D, E) or day 42 (Fig. 5f, i, j), echocardiography suggested that both ejection fraction (EF) and fraction shortening (FS) were significantly reduced after injection of DOX. On this basis, the knockout of miR-146a made the heart function more severe. HE staining showed dysfunctional or even broken myocardial fibers due to DOX, which were more serious by the deficiency of miR-146a both at day 28 (Fig. S1B) and day 42 (Fig. 5g). But the difference was more significant at day 42.

Subsequently, we extracted the protein from the ventricular muscle tissues and verified the expression of apoptosis and autophagy-related proteins in western blot. As expected, DOX treatment led to increased expression of P53, Bax and cleaved caspase-3 as well as decreased expression of Bcl-2, Beclin-1, and LC3-II/LC3-I, which were more excessive by the scarcity of miR-146a both at day 28 (Fig. S1G, H)and day 42 (Fig. 6a, c). Compared with the control group, TUNEL-positive cardiomyocytes were more frequently observed in the DOX group and the lack of miR-146a made it more obvious both at day 28 (Fig. S1C, F) and day 42 (Fig. 5h, k). But the difference was more significant at day 42. We also observed the myocardial tissue after treatment with TEM at day 42 (Fig. 6d). The muscle fibers of the control group were neat, the mitochondria were small and arranged in order, and the sputum was smooth and clear. After DOX treatment, the muscle fibers were arranged disorderly and the mitochondria were enlarged with the broken sputum. This phenomenon was more serious after miR-146a knockout. As for autophagy, normal tissues could see more lysosomes, autophagic vacuoles, and dysfunctional mitochondria surrounded by autophagic vacuoles, whereas autophagy levels were significantly reduced after DOX intervention and further reduced in knockout mice.

In our experiments, we found that miR-146a was transiently elevated for a period of time after DOX intervention, either in vivo (Fig. 5b) or in vitro (Fig. 2b), to compensate for cardiomyocytes, and when the toxicity persisted for a while, the expression of miR-146a would decrease. We hypothesized whether the toxicity of DOX to the myocardium can be assessed by detecting the abundance of miR-146a in the blood. Therefore, we tested the concentration of miR-146a in serum before and after the injection of DOX in WT mice. Interestingly, the level of miR-146a in serum was similar to that in myocardial tissue. It rose after the injection of DOX, peaked in about three days and lasted for about a week, then slowly decreased until it was lower than the pre-intervention level (Fig. 5c).

## Discussion

Since cardiomyocytes are non-renewable cells, apoptosis is a major feature of myocardial damage. Apoptosis of cardiomyocytes is regulated by autophagy activity. However, the mechanism of the interaction remains to be determined. The basal level of autophagy maintained by cardiomyocytes ensures the physiological turnover of aging and damaged organelles^28^. Autophagy is also an adaptive response under stress conditions, but when the external stimulus is excessive, autophagy will be out of regulation and thus unable to maintain the homeostasis of cardiomyocytes^29^. The complex interaction between autophagy and apoptosis in cardiomyocytes determines the extent of apoptosis and the progression of myocardial damage^30^. In the current study, we investigated the role of apoptosis and autophagy in DOX-mediated myocardial injury in vivo and in vitro through low-dose long-term DOX intervention. The increased apoptosis and reduced autophagy induced by DOX were found which can be reversed by miR-146a through TAF9b/P53 pathway (Fig. 4h).

In previous studies, the role of autophagy in DOX-induced myocardial injury has been widely debated. Our previous studies have shown that DOX upregulated apoptosis and inhibited autophagy of cardiomyocytes through a series of pathways, such as p38MAPK/p53^5^, AMPK/mTOR/Ulk1^7^, E2F1/mTORC1, and E2F1/AMPKα2^4^, which could be antagonized by resveratrol. Cardiac cells responded to starvation or serum deprivation by up-regulation of apoptosis and autophagy, and then DOX further aggravated apoptosis but repressed autophagy. Many studies have shown that boosting autophagy before the administration of DOX protects against DOX toxicity^31–34^. Li et al.^35^ showed that the upregulation of LC3-II in the early stage of myocardial toxicity induced by DOX was caused by inhibition of autophagy flux. Tandem GFP-RFP-LC3 construct was used in neonatal ventricular myocytes, showed that DOX inhibited autophagy flux in vitro^36^. Zilinyi et al.^37^ used Metformin to restore autophagy and partially reversed the myocardial damage of DOX through AMPK pathway. However, there were still many voices believing that DOX produced myocardial toxicity by upregulating autophagy rather than inhibiting autophagy flux due to differences in dose and time of administration^38,39^. Therefore, we adopted a low-dose long-term intervention, which made the experimental conditions in vitro and in vivo in line with the clinically chronic DOX-induced cardiomyopathy patients. Interestingly, we found that in the acute phase, cellular autophagy was upregulated in response to the protective mechanism of cellular toxicity, and when the pressure is excessive, autophagy flux became decompensated and decreased, and the cell loses its ability to clear cellular contents, further promoting cell apoptosis. This was consistent with most other studies^40,41^.

As for the role of miR-146a, as early as 2010, Takahiro et al. stated that in acute DOX-induce cardiotoxicity, miR-146a further aggravated DOX-mediated myocardial apoptosis by targeting ErBb4, and inhibition of miR-146a or overexpression of ErBb4 could improve this phenomenon^22^. The important role of NRG-1/ErbB signaling in heart development has been proposed very early^42,43^. As a receptor for NRG-1, ErBb4 does play an important role in this. However, with the overexpression of miR-146a, although the expression of ErBb4 was decreased, the expression of NRG-1 was significantly upregulated, which made the conclusion biased. And in the acute model, the upregulation of miR-146a was highly likely to be compensatory protection rather than pathogenic^17,44,45^. A recent study had shown the protective effect of miR-146a in diabetic glomerulopathy by targeting ErBb4^46^. Milano et al.^47^ demonstrated that cardiac progenitor cell-derived exosomes significantly improved cardiac injury induced by DOX, while miR-146a was highly enriched in these exosomes and played an important role in myocardial protection. Thus, we rethought this issue. miR-146a^−/−^ mice were used to clarify its role in myocardial injury induced by DOX. The phenotype displayed that heart function further declined with the knockout of miR-146a, as well as the increased apoptotic rate. The expression of miR-146a was rose in the acute phase and then fell, fully illustrated a process of compensatory increase and subsequent decompensation.

TAF9b was discovered as a subunit of the transcriptional regulatory polyprotein complex^26^, subsequently proved to play an important role in neurodevelopmental and mental disorders^25,48^. But as a coactivator that stabilized the structure of P53, it also participates in P53-mediated apoptosis and cell cycle regulation^24,26^. After bioinformatics analysis and validation of the dual luciferase reporter gene, TAF9b entered our study as a target gene of miR-146a. By inhibiting its activity, miR-146a can downregulate the expression of P53 and played an anti-apoptotic role. However, the relationship between decreased expression of P53 and enhanced autophagy flux remains to be explored. Studies have shown that P53 has a dual role in autophagy^23^. P53 can cause autophagy as a protection against adverse growth conditions, keeping cells still until conditions improve^49^, but on apoptosis-sensitive cells, P53 activation reduces glycolysis, increases superoxide levels, and inhibits autophagy^50^. Johnson et al. stated that DOX-induced cardiotoxicity and subsequent apoptosis, but co-treatment with Aspalathin inhibited P53 in H9c2 cardiomyocytes, resulting in decreased apoptosis and increased autophagy. The increased autophagy was associated with the activation of AMPK^34,37,51^. Another study suggested that wild-type cytoplasmic P53 may inhibit autophagy via protein-protein interactions^52^. While TAF9b only regulates P53 at the protein level^24^, so this is also an explanation for our conclusion.

As research progresses, more and more miRNAs were developed as biomarkers for disease^53,54^. Rigaud et al.^55^ showed that circulating miR-1 played like a potential biomarker of DOX-induced cardiotoxicity in breast cancer patients. There was no clinical trial to verify the relationship between serum miR-146a and DOX myocardial injury. Thus, we collected serum from mice after DOX treatment and found a compensatory up-regulation of miR-146a in the acute phase. But this still required more clinical trials to verify.

Our current research has some limitations that are worth mentioning. First, the AC16 cell line used in this study, although possessing some characteristics of cardiomyocytes, do not fully conform to primary cardiomyocytes. Besides, the miR-146a^−/^^−^ mice we used were not cardiomyocytes specific knockout, which reduced the reliability of results. In addition, in this experimental model, the relationship between P53 and autophagy needs further exploration.

To sum up, miR-146a is a protective factor in DOX-induced cardiotoxicity. It reduced the stability of P53 by targeting TAF9b, thereby reducing the apoptosis of cardiomyocytes and improving the autophagic disorder.

## Supplementary information

Supplemental material

Figure S1
