# Supplementary material for: miR-146a attenuates apoptosis and modulates autophagy by targeting TAF9b/P53 pathway in doxorubicin-induced cardiotoxicity
Source: Cell Death Dis. 2019 Sep 11;10(9):668. doi: 10.1038/s41419-019-1901-x (PMC6739392; doi:10.1038/s41419-019-1901-x)
Supplement: Supplementary file 1 — Supplemental material [file 41419_2019_1901_MOESM1_ESM.docx]

**Supplementary data**

**miR-146a attenuates apoptosis and modulates autophagy by targeting TAF9b/P53 pathway in doxorubicin-induced cardiotoxicity**

Jian-an Pan^1^, Yong Tang^2^, Jian-ying Yu^1^, Hui Zhang^1^, Jun-feng Zhang^1^, Chang-qian Wang^1^, Jun Gu^1*^

**Supplemental table1. Sequences for miR-146a mimic, mimic control, inhibitor, and inhibitor control.**

| **siRNA** | **Senses 5'---3'** |
| --- | --- |
| **miR-146a mimic** | UGAGAACUGAAUUCCAUGGGUU |
| **mimic control** | UUUGUACUACACAAAAGUACUG |
| **miR-146a inhibitor** | AACCCAUGGAAUUCAGUUCUCA |
| **inhibitor control** | CAGUACUUUUGUGUAGUACAAA |

**Supplemental table2. Sequences for siTAF9b.**

| **siRNA** | **Senses 5'---3'** |
| --- | --- |
| **siTAF9b 1** | GCAAGAUAACGCCUCCCAATT |
| **siTAF9b 2** | GCUUUCCGUUAUGUGACUATT |
| **siTAF9b 3** | CCAUGCUAAGAAACCUAAUTT |
| **Negative control** | UUCUCCGAACGUGUCACGUTT |

**Supplemental table3. Primers for real-time PCR detection.**

| **Primers** | **Sequences 5'---3'** |
| --- | --- |
| TAF9b-M-Forward Primer | CGCAAGAGAAAATGGAGCCG |
| TAF9b-M-Reverse Primer | CATCGTCCAGAATTGTAGTCACA |
| TAF9b-H-Forward Primer | GGACCTAGACTGCCACCTGA |
| TAF9b-H-Reverse Primer | CACCGTTTGTGGGGTTGCTAT |
| GAPDH-M-Forward Primer | AGGTCGGTGTGAACGGATTTG |
| GAPDH-M-Reverse Primer | TGTAGACCATGTAGTTGAGGTCA |
| GAPDH-H-Forward Primer | GGCTGCTTTTAACTCTGGTA |
| GAPDH-H-Reverse Primer | CTTGACGGTGCCATGGAATT |

The primers for miR-146a, cel-miR-39, U6 small nuclear RNA were obtained from RioboBio Company (Guangzhou, China). The sequences are covered by a patent.

**Supplemental Figure 1**. Knockout of miR-146a aggravated DOX-induced myocardial injury in vivo at day 28.

**A** Typical echocardiography at day 28 showed that DOX significantly reduced cardiac function which was more severe after miR-146a knockout. Ejection fraction (EF) and fraction shortening (FS) were shown in **D** and **E** (n=5). **B** Hematoxylin-eosin (HE) staining was used to assess myocardial damage at day 28. Scale bar indicated 20μm. **C** TUNEL staining analysis was used to detected nuclear fragmentation at day 28. Scale bar indicated 50μm. And the percentage of TUNEL positive cells in each group according to Hoechst nuclear staining was indicated in **F** (n=5). **G** Related proteins of myocardial tissue were detected by western blot after DOX intervention at day 28 and the relative protein expressive were determined normalized to GAPDH in **H** (n=5). * P<0.05, ** P<0.01, *** P<0.001.
