# Supplementary figures and images for: miR-146a attenuates apoptosis and modulates autophagy by targeting TAF9b/P53 pathway in doxorubicin-induced cardiotoxicity
Source: Cell Death Dis. 2019 Sep 11;10(9):668. doi: 10.1038/s41419-019-1901-x (PMC6739392; doi:10.1038/s41419-019-1901-x)

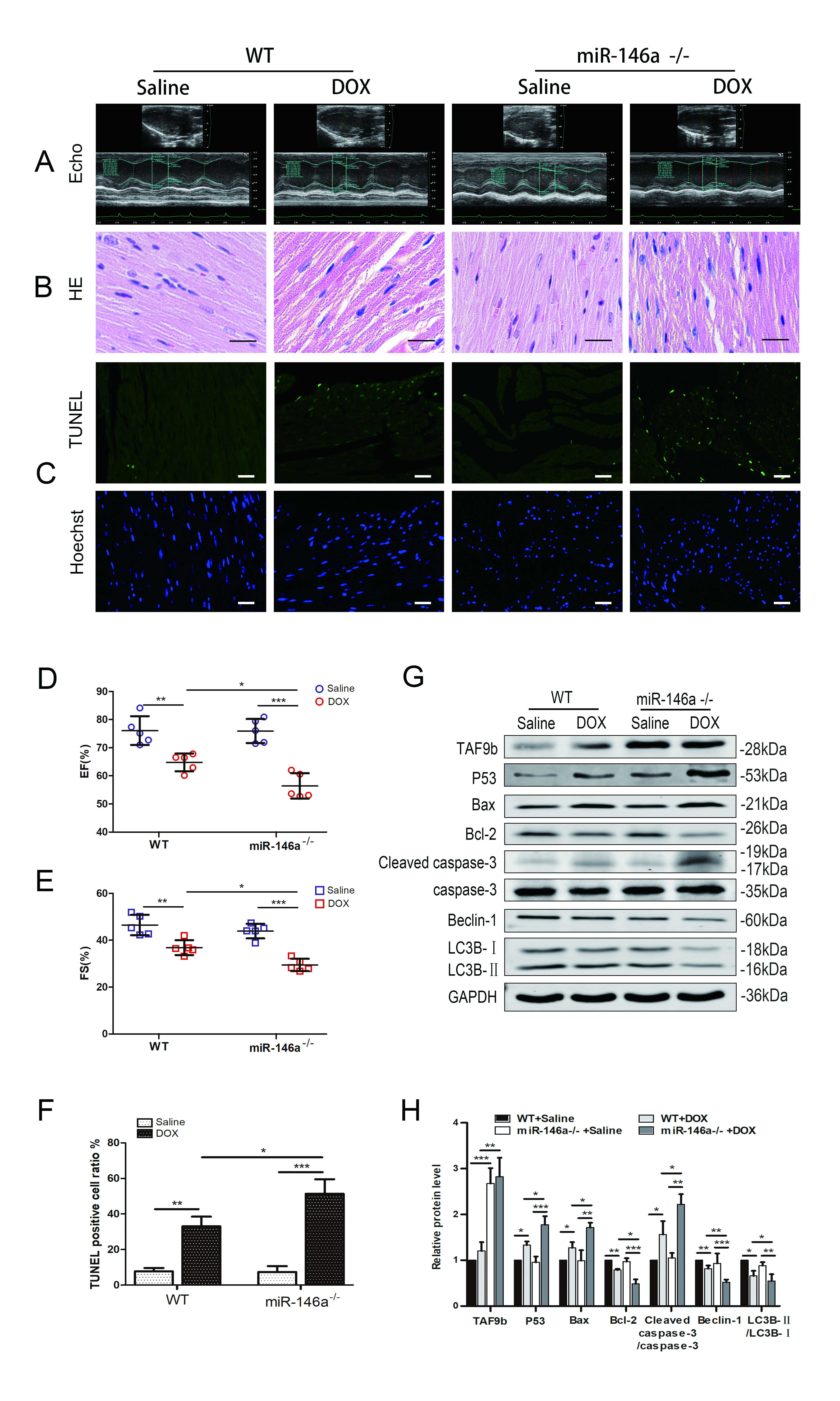

Supplement: Supplementary file 2 — Figure S1 [file 41419_2019_1901_MOESM2_ESM.tif]
